# Sulfoxaflor and Flupyradifurone: Efficacy, Residue Dynamics, and Dietary Risk Assessment in *Cudrania tricuspidata*

**DOI:** 10.3390/toxics14020117

**Published:** 2026-01-26

**Authors:** Junheon Kim, Eunji Yu

**Affiliations:** Forest Entomology and Pathology Division, National Institute of Forest Science, Seoul 02455, Republic of Korea; yueunjio@korea.kr

**Keywords:** *Aphis citricidus*, *Lycorma delicatula*, pre-harvest interval, risk assessment, *Maclura tricuspidata*

## Abstract

This study explored the efficacy, residue dynamics, and dietary risks of sulfoxaflor and flupyradifurone in *Cudrania tricuspidata*. Following two applications, residue levels of sulfoxaflor and flupyradifurone decreased from 0.254 to 0.012 mg/kg and 0.732 to 0.016 mg/kg, respectively, over a period of 22 days. The half-lives (*t*_1/2_) in fruits and leaves ranged from 7.0 to 13.6 days. LC-MS/MS analysis showed recovery rates of 79.8–94.9% and RSD < 8.5%. Both pesticides effectively controlled hemipteran pests, reducing aphid and spotted lanternfly populations by >90%. Acute and chronic dietary risk assessments indicated acute hazard index (aHI) and chronic hazard quotient (HQ) values remarkably < 1, suggesting a negligible health risk. According to these results, sulfoxaflor and flupyradifurone have recently been registered as pesticides for *C. tricuspidata* against hemipteran pests, with a recommended pre-harvest interval of 7 days, as projected residue levels (0.078–5.213 mg/kg) were below established maximum residue limits (MRLs). These findings indicate a low dietary risk associated with sulfoxaflor and flupyradifurone in *C. tricuspidata* when applied according to the evaluated application rates and pre-harvest interval.

## 1. Introduction

*Cudrania tricuspidata*, commonly referred to as *Maclura tricuspidata* (Carr.) Bur., which belongs to the Moraceae family, is a deciduous tree characterized by broad leaves and thorny branches. This species naturally inhabits and is cultivated across several East Asian countries, including Korea, China, Japan, and Vietnam, as well as in the USA [1,2].

*C. tricuspidata* has been an integral component of traditional herbal medicines in Eastern countries for many years [3,4]. Recent studies have identified its extensive bioactive properties, revealing a range of biological activities including anticancer, antioxidant, anti-inflammatory, anti-obesity, and antibacterial effects, highlighting its therapeutic efficacy [4,5,6]. These diverse effects have facilitated their incorporation into functional health-food supplements. In Korea, *C. tricuspidata* is consumed in various forms such as fresh fruits, juice, jam, and tea powders. The use of fruit and leaves as dietary supplements and functional food ingredients has been actively pursued in multiple sectors [4,5].

The fruits and leaves of *Cudrania tricuspidata* are susceptible to damage by several insect pests, including hemipteran insects such as aphids and planthoppers, as well as leaf beetles and lepidopteran larvae (e.g., *Euchera nana*, saturniid, and pyralid caterpillars) [7].

Among these pests, the aphid *Toxoptera citricidus* (syn. *Aphis citricidus*; Hemiptera: Aphididae) and the spotted lanternfly *Lycorma delicatula* (Hemiptera: Fulgoridae) were frequently observed causing damage to *C. tricuspidata* during preliminary field observations. *T. citricidus* has been previously reported as a pest of *C. tricuspidata* [8], whereas no formal documentation exists regarding damage to *C. tricuspidata* caused by *L. delicatula*. Aphids damage host plants by extracting phloem sap from leaves, which can result in leaf desiccation and promote the development of sooty mold due to the accumulation of honeydew excretions [9]. *L. delicatula* predominantly attaches to fruits, where it pierces the tissue and feeds on sap, leading to direct fruit damage [10]. Based on their frequent occurrence and distinct damage characteristics observed in the field, these two hemipteran pests were selected as target species for pesticide-based control. Chemical control remains one of the most commonly applied strategies for mitigating damage caused by insect pests.

Effective pest management requires the application of appropriate insecticides at optimal times and in recommended quantities. Misuse of chemical insecticides may prove ineffective, whereas excessive application poses significant health risks owing to residue accumulation. Inappropriate pesticide application during the fruiting phase and disregard for pre-harvest intervals can result in residual pesticide accumulation in the edible plant parts. Currently, there are no registered pesticides for *C. tricuspidata* against insect pests, particularly hemipteran pests. Therefore, it is imperative to register an appropriate pesticide and conduct residual analysis to establish safety protocols for its application [11]. This study investigated the efficacy and residue levels of two pesticides, sulfoxaflor and flupyradifurone, in the fruit and leaves of *C. tricuspidata*. Both sulfoxaflor and flupyradifurone are classified as nicotinic acetylcholine receptor (nAChR) competitive modulators (IRAC group 5), belonging to the sulfoximine and butenolide classes, respectively [12]. Sulfoxaflor is a novel insecticide that is effective against sap-feeding insects [13,14], whereas flupyradifurone has demonstrated efficacy against hemipteran pests [13,15,16]. Consequently, these pesticides were selected for registration on the basis of their effectiveness against hemipteran pests.

The residue characteristics of sulfoxaflor and flupyradifurone have been widely reported for various conventional food crops. The residue behavior and dietary risk assessment of sulfoxaflor and flupyradifurone have been documented in vegetables and fruits such as cucumber, tomato, cabbage, grape, olive, and apple [17,18,19,20,21], and in agricultural commodities including pistachio, date, eggplant, ginseng, and pepper [22,23,24,25], respectively. These field residue trials and processing studies have shown that both insecticides exhibit crop- and environment-dependent dissipation patterns, and that certain metabolites, such as 6-chloronicotinic acid, may persist depending on the crop matrix.

While these previous studies were largely focused on residue behavior and dietary risk assessment, the actual insecticidal efficacy of the pesticides under field conditions was not evaluated. Consequently, no field-based information is available on the evaluation of pesticidal efficacy combined with residue behavior and dietary risk assessment of the pesticides in *Cudrania tricuspidata*. Importantly, evaluating pesticidal efficacy together with residue behavior and dietary risk assessment is essential for interpreting the practical relevance of residue data and for establishing scientifically sound pre-harvest intervals under actual field conditions.

Therefore, this study aimed to assess the pesticidal efficacy of sulfoxaflor and flupyradifurone against hemipteran insect pests, validate a method for the quantification of these pesticides and their metabolites in *C. tricuspidata*, and conduct a dietary risk assessment.

## 2. Materials and Methods

### 2.1. Study Area and Characteristics of C. tricuspidata

This study was conducted to evaluate pesticide residues in a *C. tricuspidata* plantation located in Buyeo, Republic of Korea (36°11′ N, 126°49′ E), starting from Mid-August. The plantation comprised 13-year-old trees. The mean height and canopy width (in meters) of *C. tricuspidata* were 3.00 ± 0.32 and 4.23 ± 0.33 (±SD) for sulfoxaflor, and 3.13 ± 0.41 and 4.38 ± 0.46 (±SD) for flupyradifurone. Pesticide efficacy trials were conducted in Daegu (35°59′ N 128°38′ E) and Seonngju (35°55″ N 128°16′ E), Gyeongsangbukdo, Republic of Korea.

### 2.2. Pesticides, Chemicals, and Reagents

**Figure 1:**
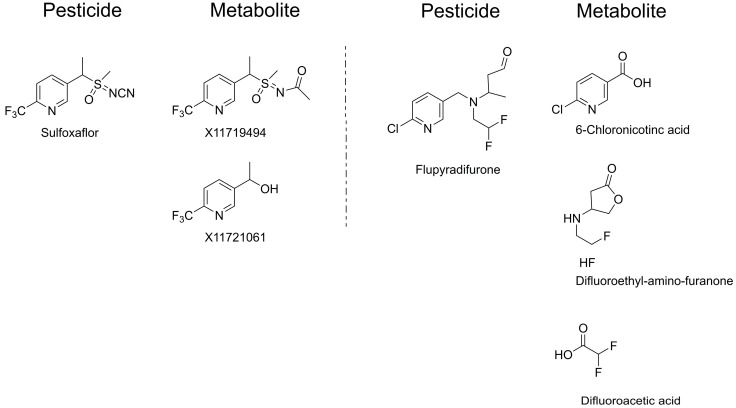
Structures of pesticides and their metabolites.

The pesticides used for evaluating residue levels and efficacy, namely, sulfoxaflor water-dispersible granule (WG) 7% and flupyradifurone soluble concentrate (SL) 17.09%, were sourced from a commercial distributor. Pesticide standards for sulfoxaflor (99.7% pure), X11719474 (99.5% pure), and X11721061 (87.0% pure), as well as those for flupyradifurone (99.4% pure), 6-chloronicotinic acid (98.9% pure), difluoroethyl-amino-furanone (98.5% pure), and difluoroacetic acid (95.5% pure) (Bayer AG), were purchased from Dow AgroSciences (Indianapolis, IN, USA) and Bayer AG (Leverkusen, Germany), respectively (Figure 1). Stock solutions of each pesticide standard were prepared at a concentration of 1000 μg/mL, using either acetonitrile or acetone as the solvent. For the sulfoxaflor analysis, an Envi-Carb SPE cartridge (250 mg/6 mL; Supelco, St. Louis, MO, USA) was employed as the dispersive solid-phase extract (SPE), whereas for flupyradifurone, an HL8 SPE cartridge (200 mg/6 mL; Waters, Milford, MA, USA) was used. Acetonitrile, methanol, and water were procured from J.T. Baker (Philipsburg, NJ, USA), and formic acid was obtained from Sigma-Aldrich (St. Louis, MO, USA).

### 2.3. Pesticide Application and Sample Preparation for Residue Analysis

Each pesticide was diluted to a 2000-fold concentration, corresponding to the recommended dosage, and applied twice to *C. tricuspidata* at weekly intervals at a volume of 500 L/10 a (=100 m^2^). This resulted in a total application of active ingredient (a.i.) amounting to 0.035 kg a.i./10 a for sulfoxaflor and 0.086 kg a.i./10 a for flupyradifurone for pesticide residue analysis. Fruit and leaf samples were harvested at specified intervals: 0 days (2 h post-spraying), and subsequently at 8, 15, and 22 days following the second application. The samples were sealed in sampling bags and stored at −20 °C prior to analysis to prevent pesticide degradation. To estimate the dietary intake in plant commodities, the residue definition for sulfoxaflor and flupyradifurone was defined as the sum of sulfoxaflor and its metabolites (X11719474 and X11721061), expressed as total sulfoxaflor, and the sum of flupyradifurone and its metabolites (6-chloronicotinic acid, difluoroethyl-amino-furanone, and difluoroacetic acid), expressed as total flupyradifurone (Figure 1). Consequently, the total residues were calculated based on molecular weight [25].

### 2.4. Sample Extraction for Analysis

In September, a substantial quantity of *C. tricuspidata* was collected, comprising over 1 kg of fruits and 500 g of leaves. Then, 50 g of fruit and 10 g of leaves were crushed. For sulfoxaflor analysis, crushed samples were extracted using 100 mL methanol and 1 mL formic acid over a period of 40 min. Alternatively, for flupyradifurone analysis, the samples were extracted with 100 mL acetonitrile (ACN:water, 80:20) and 1 mL formic acid and agitated at 180 rpm before filtration. The resulting filtrate was rinsed with 30 mL of methanol, and the methanol extracts were subsequently combined. For sulfoxaflor analysis, an SPE cartridge was conditioned with 3 mL of methanol, and a 2 mL sample dissolved in 200 mL of methanol was loaded. The cartridge was eluted with 5 mL methanol, followed by nitrogen evaporation to dryness. For flupyradifurone analysis, an SPE cartridge was conditioned with 5 mL of ACN, and a 2 mL sample dissolved in 200 mL of ACN: water was loaded. The cartridge was eluted with 6 mL of 1% formic acid in ACN, followed by nitrogen evaporation to dryness, and the resulting dried residue was reconstituted in 1 mL of methanol or ACN:water (8:2) for LC/MS/MS analysis. All experiments were conducted in triplicate for each period.

### 2.5. Liquid Chromatography-Tandem Mass Spectrometer (LC-MS/MS) Analysis

Pesticide residue analysis was performed at AB solution Inc. (Hwasung, Gyeonggi, Republic of Korea) using an LC/MS-8040 system equipped with a triple quadrupole mass detector (Shimadzu, Kyoto, Japan). Pesticide separation was achieved using a Capcellpak C18 MG III column (150 mm × 2.0 mm, 5 μm particle size; Osaka Soda, Amagasaki, Hyogo, Japan) for sulfoxaflor and its metabolites, a Hypersil gold C18 column (150 mm × 2.1 mm, 5 μm particle size; ThermoFisher, Waltham, MA, USA) for flupyradifurone and its metabolites with the exception of difluoroacetic acid, which was analyzed using a PC HILIC column (50 mm × 2.0 mm, 5 μm particle size; Osaka Soda, Osaka, Japan). The specific analytical conditions for each pesticide are detailed in Supplementary Material (Tables S1–S3). The column temperature was maintained at 40 °C, and the injection volume was set at 3 μL. The electrospray ionization (ESI) source was operated in both positive and negative ionization modes, with an ion spray voltage of 4500 V and a heat block temperature of 400 °C. MS data were acquired in the multiple reaction monitoring (MRM) mode, with specific parameters for each pesticide outlined in Table 1.

### 2.6. Preparation of Standard and Matrix-Matched Standard Solutions

In a 100 mL volumetric flask, 0.0100 g of each pesticide standard was weighed using an electronic balance (Ohaus Instruments, Parsippany, NJ, USA). The primary stock solutions were formulated by dissolving the standards in methanol for sulfoxaflor and in ACN for flupyradifurone. The concentration was calculated using the following formula [26]: standard solution (µg/mL) = (pesticide standard (mg) × purity (%) × 1000)/(final volume (mL) × 100). Working standard solutions were prepared by serial dilution of stock solutions. Matrix-matched standard solutions were prepared using the matrix blanks obtained from the extraction and purification methods described in Section 2.4. The final concentrations of sulfoxaflor, flupyradifurone, and their metabolites in standard solutions were 0.05, 0.1, 0.2, 0.5, 1.0, and 2.0 mg/L. Chromatograms of each pesticide standard are presented in the Supplementary Material (Figure S1).

### 2.7. Method Validation

In accordance with the Korea Food Code guidelines, the analytical method was comprehensively validated by assessing key parameters including linearity, accuracy, limits of detection (LOD), limit of quantification (LOQ), and recovery rates [27].

Calibration curves were evaluated using standard solutions prepared as described in Section 2.5, along with matrix-matched standard solutions at six calibration points: 0.05, 0.1, 0.2, 0.5, 1.0, and 2.0 mg/L. The linearity was determined by calculating the correlation coefficient (*R*^2^) from the calibration points. The calibration curves were generated by plotting the detector response (peak area) against the corresponding concentration. Linear regression equations and *R*^2^ were derived to analyze the relationship between the pesticide concentration and peak area (Supplementary Material; Table S4). The LOD and LOQ were statistically determined as 3σ and 10σ, respectively, with the standard deviation (σ) calculated from seven replicate measurements of the samples spiked at the LOQ level. To determine the recovery rate, 1 mL of pesticide standard solution at concentrations of 5 and 25 mg/L was added to 50 and 25 g of untreated fruit and leaf samples, respectively. The mixture was homogenized and incubated for 30 min, followed by sample analysis, according to the procedure described in Section 2.4. All experiments were performed in triplicate.

### 2.8. Risk Assessment

To evaluate short-term (acute) dietary risk to consumers, the acute hazard index (aHI) was derived by comparing the estimated short-term intake (ESTI; mg/kg body weight (b.w.)/day) with the acute reference dose (ARfD; mg/kg b.w./day). According to the European Commission [28], the ARfD values for sulfoxaflor and flupyradifurone are 0.25 and 0.15 mg/kg b.w./day, respectively. For long-term (chronic) dietary risk assessment, the hazard quotient (HQ) was calculated using the estimated daily intake (EDI; mg/kg b.w./day) and acceptable daily intake (ADI; mg/kg b.w./day). The ADI values for sulfoxaflor and flupyradifurone were set at 0.05 and 0.078 mg/kg b.w./day, respectively, by the Korean Ministry of Food and Drug Safety [29], and 0.04 and 0.064 mg/kg b.w./day, respectively, by the European Commission [28]. To enhance the precision of risk estimation, both ARfD and ADI values were expressed as daily intake for a standard 60 kg adult. In the absence of specific national dietary intake data for *C. tricuspidata*, default intake amounts were applied based on the manufacturer’s recommendations: 9 g/day for fruit powder, 300 g/day for fruit extract, and 9 g/day for leaves. Each intake represents a conservative worst-case scenario derived from manufacturer-recommended preparation volumes and amounts due to lack of consumer survey data.

Risk was deemed acceptable when both the aHI and HQ values were below 1, indicating no significant health concerns. Conversely, values exceeding 1 were interpreted as posing a potential risk to human health. Higher aHI and HQ values suggest higher levels of acute and chronic exposure [30].

The relevant formulae are as follows:

ESTI = the highest residue level (mg/kg) × food consumption (g/day)/body weight (kg b.w.)(1)

aHI = ESTI/ARfD (mg/kg b.w./day) (2)

EDI = mean residue level (mg/kg) × food consumption (kg/day)/body weight (kg b.w) (3)

HQ = EDI/ADI (4)

### 2.9. Evaluation of Pesticide Efficacy Against Aphid and Spotted Lanternfly

The efficacy of pesticides against aphids and spotted lanternflies was assessed in May. Prior to pesticide application, the aphid populations were quantified, confirming that each replicate exceeded 100 individuals. Post-pesticide application, the branches were enclosed in nets to prevent insects from escaping. For the spotted lanternfly test, 60 nymphs were introduced onto the branches and leaves of *C. tricuspidata*, which were then covered with a net to ensure containment, and the pesticide was applied to the hemipteran insect pest in the net. This method was used to ensure uniform exposure while preventing insect escape under semi-field conditions. The survival rate of the insects was assessed after seven days. Each pesticide was diluted 2000-fold and administered twice with a one-week interval, to *C. tricuspidata* at a dosage of 500 L/10 a, resulting in an a.i. of 0.035 kg a.i./10 a of sulfoxaflor and 0.086 kg a.i./10 a of flupyradifurone. As a control, only water without pesticides was used. Experiments were conducted in triplicate at each location (Daegu and Seongju). The control efficacy was calculated based on Abbott’s mortality [31].

### 2.10. Data Analysis

The variations in residue levels of total sulfoxaflor and flupyradifurone over time post-application were analyzed using one-way analysis of variance (ANOVA) followed by a post hoc comparison employing Tukey’s honest significant difference (HSD) at a 5% error rate. The half-life (*t*_1/2_) of the insecticide was calculated using Equation (5), where *k* denotes the slope of the regression line [32]. The regression equation was derived by plotting the residue amount against the days following pesticide application.

(5) $$t_{1/2}=\frac{ln2}{k}$$

Statistical analysis was performed using JMP software ver. 9.0.2 (SAS Institute, Cary, NC, USA).

## 3. Results

### 3.1. Analytic Validation

The analyte recovery rates of sulfoxaflor and flupyradifurone from fruits ranged from 85.1–94.9% and 80.3–91.2%, while those from leaves ranged from 82.5–92.3% and 79.8–91.0%, respectively. The relative standard deviation (%RSD) of sulfoxaflor and flupyradifurone from fruit was 1.98–6.28% and 1.75–9.66%, and those from leaves were 0.84–3.29% and 0.49–2.29%, respectively, meeting the criteria for quantitative methods [33]. The LOQ (mg/kg) and LOD (mg/kg) for sulfoxaflor and flupyradifurone from fruits were 0.01 and 0.01, and from leaves were 0.05 and 0.01, respectively. The validation parameters of the analytical method for the pesticide residues are listed in Table 2.

### 3.2. Pesticide Residues

The pesticide residue levels were influenced by the type of pesticide used, duration of exposure, and the specific plant parts analyzed (Table 3 and Table 4). Typically, pesticide residue levels were higher in leaves than in fruits and consequently declined over time, post-spraying. Sulfoxaflor and flupyradifurone residue levels in fruits following 0-day application were 0.153 and 0.723 mg/kg, respectively, decreasing to 0.060 and 0.280 mg/kg, respectively, 8 days post-application. In leaves, after 0-day application, the residue levels were 2.173 and 8.923 mg/kg, decreasing to 0.310 and 4.157 mg/kg, respectively, after 8 days of application. No significant differences in residue levels were observed in fruits and leaves 8, 15, and 22 days post-application, irrespective of the pesticide used. The regression equations are presented in the Supplementary Materials (Figure S2). The *t*_1/2_ of sulfoxaflor in fruits and leaves was 10.78 and 3.84 days, respectively, while that of flupyradifurone was 14.02 and 12.96 days, respectively.

### 3.3. Risk Assessment for the Detected Residues

The short-term (acute) and long-term (chronic) risks associated with pesticide consumption through fruits and leaves at three distinct post-application intervals are detailed in Table 5. The aHI and HQ values for all pesticides tested were significantly below the established safety threshold. Both short- and long-term risk evaluations indicated a decline in ESTI and EDI values as the duration post-application increased. The risk of consuming fruit extracts was greater than that of consuming fruit and leaf powders. At eight days post-application, the risks were minimal, suggesting negligible short-term and chronic risks from pesticide exposure through the consumption of fruits and leaves.

### 3.4. Pesticide Efficacy Against Aphid and Spotted Lanternfly

Pesticide efficacy results are presented in Table 6. The trials were executed at two different sites, and a preliminary two-way ANOVA indicated no significant site effect and site × treatment interaction (treatment: *p* < 0.05, Site: *p* > 0.05, site × treatment: *p* > 0.05). As a result, the data from both sites were consolidated. The pesticidal efficacies of sulfoxaflor WG against aphid and spotted lanternfly were consistently 100%, while those of flupyradifurone SL were 91.9% and 96.0%, respectively, satisfying the registration criteria for pesticide (>90%) [11].

## 4. Discussion

This study provides evidence that sulfoxaflor WG (7%) and flupyradifurone SL (17.09%) are potent pesticides for the control of aphids and spotted lanternflies. The residual levels of these pesticides were assessed and found to be within acceptable limits (aHI < 1, HQ < 1), indicating negligible risk to consumers. Consequently, the Korea RDA has officially approved these pesticides for controlling aphids and spotted lanternflies, recommending a 7-day pre-harvest interval as a safety guideline [34]. Additionally, the Korean Ministry of Food and Drug Safety (MFDS) has established maximum residue limits (MRL) for sulfoxaflor at 1.5 mg/kg in fruits and for flupyradifurone at 1.5 and 10.0 mg/kg in fruits and leaves, respectively [29].

These findings indicate that the pesticide residue levels in the fruits and leaves of *C. tricuspidata* were initially high, but decreased over time. Importantly, the *t*_1/2_ of sulfoxaflor and flupyradifurone in the fruits and leaves of *C. tricuspidata* was longer than that in other crops.

The *t*_1/2_ of pesticides is subject to variations based on the type of crop and regional environment (Table 7). For sulfoxaflor, the *t*_1/2_ has been documented as 1.6–2.9 days in cucumber [14], 3.87 days in spinach, 3.54 days in Korean cabbage [21], and 14.20 and 4.18 days in the fruits and cladodes, respectively, in prickly pear [35]. For flupyradifurone, the *t*_1/2_ was reported as 4.5–7.9 days in ginseng plants [25] and 1.6 days in tea [16]. These discrepancies can be attributed to a range of biotic and abiotic factors such as microbial decomposition, weathering, heat, and sunlight (photodegradation) [26]. Despite the prolonged *t*_1/2_ in *C. tricuspidata*, residue regression equations predicted that after a 7-day pre-harvest interval, sulfoxaflor residue levels would be 0.078 mg/kg in fruits and 0.546 mg/kg in leaves, whereas that of flupyradifurone was 0.412 mg/kg in fruits and 5.213 mg/kg in leaves. These concentrations were all within the MRL for sulfoxaflor and flupyradifurone, as established by the Korean Ministry of Food and Drug Safety [29]. Consequently, a 7-day pre-harvest interval is considered a prudent and safe guideline for the application of these pesticides in *C. tricuspidata*.

The findings indicated an aHI and HQ < 1 for the fruits and leaves of *C. tricuspidata* when consumed in the form of tea powder or beverage extracts. An HQ < 1 suggests a low safety risk to human health from a specific pesticide, thereby classifying food as safe for consumption. Conversely, an HQ > 1 signifies an unacceptable safety risk, implying potential adverse health impacts and rendering the food unsafe for consumption [32,36]. The current study confirmed the safety of *C. tricuspidata* powder and extracts for consumption. For the risk assessment, the default intake amounts were derived from the manufacturer’s recommendations. However, these amounts may overestimate actual consumption, as consumers typically do not consume fruit and leaf powder three times daily. Consequently, the actual safety risks to human health posed by pesticides may be significantly lower than the estimated values.

Food processing techniques play a crucial role in determining the pesticide residue levels in agricultural products. Among these techniques, drying is particularly important because of its potential to cause varying rates of pesticide degradation contingent upon the specific compound and drying conditions of fruits [37,38]. Often, the drying process can lead to an increase in the pesticide residue concentration as a result of moisture evaporation [39,40]. Evaluating the effect of drying on pesticide residue levels is essential, considering that *C. tricuspidata* fruits and leaves are frequently processed into a powder form through drying. The processing factor (PF), defined as the ratio of residue levels in processed products to those in raw agricultural commodities, is a critical parameter for these evaluations. PFs are indispensable for the accurate assessment of dietary exposure, particularly when the processed form is the primary mode of consumption. Although this study did not investigate PFs, future research should incorporate them to better simulate real-world exposure scenarios and ensure the safety of dried *C. tricuspidata* products.

This study analyzed sulfoxaflor and flupyradifurone residues using a liquid–liquid extraction (LLE) technique in conjunction with SPE in accordance with the Korea Food Code [27]. Despite the recent adoption of the Quick Easy Cheap Effective Rugged Safe (QuEChERS) method by the Korean government for multi-residue pesticide analysis, it has not been officially adopted for single-residue analysis of pesticides such as sulfoxaflor and flupyradifurone. A comparative study by Shin et al. [41] indicated that the QuEChERS method achieves higher recovery rates than the LLE-SPE approach. Additionally, prior research has demonstrated the successful application of QuEChERS for both single- and multi-residue pesticide analyses [17,42,43]. Although existing methodologies comply with regulatory standards, future research should focus on optimizing QuEChERS-based techniques to improve the detection efficiency and effectiveness of sulfoxaflor and flupyradifurone residues in the fruits and leaves of *C. tricuspidata*.

## 5. Conclusions

This study provides the first field-based assessment of the efficacy, residue dynamics, and dietary risk of sulfoxaflor and flupyradifurone in *Cudrania tricuspidata*. Both insecticides showed high control efficacy against *Aphis citricidus* and *Lycorma delicatula*, with control efficiency exceeding 90% under the tested field conditions. Initial residue concentrations in fruits were 0.153 mg/kg for sulfoxaflor and 0.723 mg/kg for flupyradifurone, which declined to ≤0.060 mg/kg and ≤0.280 mg/kg, respectively, within 8 days after the final application. Residue levels in leaves were higher than those in fruits but exhibited a consistent dissipation trend over time.

The dissipation half-lives were estimated to range from 3.84 to 10.78 days for sulfoxaflor and from 12.96 to 14.02 days for flupyradifurone in fruits and leaves. Regression-based predictions indicated that residue levels at a 7-day pre-harvest interval would remain below the established maximum residue limits. Dietary risk assessment demonstrated that both acute hazard index (aHI) and chronic hazard quotient (HQ) values were well below 1 across all evaluated consumption scenarios, including conservative intake assumptions for fruit and leaf products.

Overall, these results indicate a low dietary risk associated with sulfoxaflor and flupyradifurone when applied according to the evaluated application rates and pre-harvest interval. The findings provide quantitative baseline data that may support future residue management and risk assessment of these insecticides in *C. tricuspidata*; however, further studies considering long-term use patterns and processing factors would be beneficial to refine exposure assessments.

## Figures and Tables

**Table 1:** Multi-reaction monitoring (MRM) parameters for sulfoxaflor and flupyradifurone.

Compound	Precursor Ion	Product Ion	Q1 Pres Bias (V)	CE (V)	Polarity	Rt (Min)
Sulfoxaflor						
Sulfoxaflor	278.00	173.85	−10	−9	Positive	7.118
	278.00	153.85	−14	−29	Positive	7.422
X11719474	296.00	104.90	−10	−17	Positive	5.077
	296.00	153.70	−14	−30	Positive	
X11721061	191.90	42.95	−13	−26	Positive	6.479
	191.90	129.70	−12	−21	Positive	
Flupyradifurone						
Flupyradifurone	288.90	98.90	−19	−53	Positive	5.350
	288.90	90.05	−20	−46	Positive	
6-Chloronicotinic acid	157.80	121.90	−10	−20	Positive	5.066
	157.80	51.00	−26	−39	Positive	
Difluoroethyl-amino-furanone	163.90	72.90	−11	−21	Positive	4.322
	163.90	64.85	−28	−31	Positive	
Difluoroacetic acid	95.00	51.00	14	14	Negative	2.911

Dwell time was 100 ms; CE collision energy. Rt: Retention time.

**Table 2:** Recovery (%), Limit of Quantification (LOQ), and Limit of Detection (LOD) for the analyzed pesticides and their metabolites.

Pesticide	Recovery (% RSD) (%, Mean ± SD, *N* = 3)		LOQ (mg/kg)	LOD (ng)
	0.1 mg/kg	0.5 mg/kg		
Fruits				
Sulfoxaflor				
Sulfoxaflor	92.3 ± 5.8 (6.28)	87.8 ± 2.8 (3.19)	0.01	0.01
X11719474	94.9 ± 4.0 (4.21)	90.7 ± 1.8 (1.98)	0.01	0.01
X11721061	94.1 ± 5.1 (5.42)	85.1 ± 3.1 (3.64)	0.01	0.01
Flupyradifurone				
Flupyradifurone	91.2 ± 1.6 (1.75)	89.3 ± 3.1 (3.47)	0.01	0.01
6-Chloronicotinic acid	80.3 ± 2.9 (3.61)	82.6 ± 2.5 (3.03)	0.01	0.01
Difluoroethyl-amino-furanone	81.8 ± 7.9 (9.66)	87.4 ± 4.9 (5.61)	0.01	0.01
Difluoroacetic acid	82.9 ± 1.7 (2.05)	83.0 ± 4.8 (5.78)	0.01	0.01
Leaves				
Sulfoxaflor				
Sulfoxaflor	92.3 ± 1.2 (1.30)	83.5 ± 0.8 (1.44)	0.05	0.01
X11719474	89.6 ± 1.0 (1.12)	83.8 ± 0.7 (0.84)	0.05	0.01
X11721061	91.1 ± 3.0 (3.29)	82.5 ± 0.7 (0.85)	0.05	0.01
Flupyradifurone				
Flupyradifurone	89.9 ± 0.7 (0.78)	80.9 ± 0.4 (0.49)	0.05	0.01
6-Chloronicotinic acid	88.7 ± 2.4 (2.71)	79.8 ± 0.7 (0.88)	0.05	0.01
Difluoroethyl-amino-furanone	90.1 ± 1.2 (1.33)	82.2 ± 7.9 (9.61)	0.05	0.01
Difluoroacetic acid	91.0 ± 1.2 (1.32)	82.9 ± 1.9 (2.29)	0.05	0.01

**Table 3:** Dissipation patterns of sulfoxaflor and flupyradifurone in fruits.

Pesticide	Residue (mg/kg, Mean ± SD, Min.–Max. *N* = 3) After Final Application, (Days) ^a^				Statistical Value
	22 d	15 d	8 d	0 d	
Sulfoxaflor (Total)	0.033 ± 0.006 b (0.03–0.04)	0.040 ± 0.010 b (0.03–0.05)	0.060 ± 0.010 b (0.05–0.07)	0.153 ± 0.035 a (0.12–0.19)	*F*_3,8_ = 25.30 *p* = 0.0002
Sulfoxaflor	0.033 ± 0.006 (0.03–0.04)	0.040 ± 0.010 (0.03–0.05)	0.060 ± 0.010 (0.05–0.07)	0.153 ± 0.035 (0.12–0.19)	
X11719474	<LOQ	<LOQ	<LOQ	<LOQ	
X11721061	<LOQ	<LOQ	<LOQ	<LOQ	
Flupyradifurone (Total)	0.227 ± 0.081 b (0.14–0.30)	0.293 ± 0.081 b (0.22–0.38)	0.280 ± 0.017 b (0.26–0.29)	0.723 ± 0.167 a (0.57–0.90)	*F*_3,8_ = 15.50 *p* = 0.0011
Flupyradifurone	0.053 ± 0.015 (0.04–0.07)	0.087 ± 0.012 (0.08–0.10)	0.133 ± 0.023 (0.12–0.16)	0.647 ± 0.020 (0.52–0.80)	
6-Chloronicotinic acid	0.017 ± 0.006 (0.01–0.02)	0.020 ± 0.000 (0.02)	0.023 ± 0.006 (0.02–0.03)	0.010 ± 0.000 (0.01)	
Difluoroethyl-amino-furanone	0.013 ± 0.006 (0.01–0.02)	0.013 ± 0.006 (0.01–0.02)	0.010 ± 0.000 (0.01)	0.010 ± 0.000 (0.01)	
Difluoroacetic acid	0.040 ± 0.020 (0.02–0.06)	0.050 ± 0.020 (0.03–0.07)	0.030 ± 0.010 (0.02–0.04)	0.017 ± 0.006 (0.01–0.02)	

^a^ Means followed by the same letter in the row are not different from each other (Tukey’s HSD test at *p* = 0.05).

**Table 4:** Dissipation patterns of sulfoxaflor and flupyradifurone in leaves.

Pesticide	Residue (mg/kg, Mean ± SD, Min.–Max. *N* = 3) at Day After Application ^a^				Statistical Value
	22 d	15 d	8 d	0 d	
Sulfoxaflor (Total)	0.11 b (0.11)	0.210 ± 0.035 b (0.17–0.23)	0.310 ± 0.044 b (0.28–0.36)	2.173 ± 0.621 a (1.63–2.85)	*F*_3,8_ = 30.88 *p* < 0.0001
Sulfoxaflor	0.11 (0.11)	0.210 ± 0.035 (0.17–0.23)	0.310 ± 0.044 (0.28–0.36)	2.173 ± 0.621 (1.63–2.85)	
X11719474	<LOQ	<LOQ	<LOQ	<LOQ	
X11721061	<LOQ	<LOQ	<LOQ	<LOQ	
Flupyradifurone (Total)	2.703 ± 0.335 b (2.44–3.08)	3.020 ± 0.156 b (2.92–3.20)	4.157 ± 0.708 b (3.34–4.60)	8.923 ± 1.145 a (7.80 ± 10.09)	*F*_3,8_ = 43.90 *p* < 0.0001
Flupyradifurone	1.880 ± 0.132 (1.78–2.03)	2.18 ± 0.101 (2.09–2.29)	2.553 ± 0.493 (1.99–2.91)	8.06 ± 2.029 (6.55–9.38)	
6-Chloronicotinic acid	0.067 ± 0.015 (0.05–0.08)	0.093 ± 0.023 (0.08–0.12)	0.130 ± 0.028 (0.11–0.15)	0.065 ± 0.007 (0.06–0.07)	
Difluoroethyl-amino-furanone	<LOQ	<LOQ	<LOQ	<LOQ	
Difluoroacetic acid	0.233 ± 0.070 (0.16–0.30)	0.223 ± 0.071 (0.16–0.30)	0.480 ± 0.036 (0.45–0.52)	0.227 ± 0.067 (0.17–0.30)	

^a^ Means followed by the same letter in the row are not different from each other (Tukey’s HSD test at *p* = 0.05).

**Table 5:** Risk assessment of sulfoxaflor and flupyradifurone according to formulation.

Formulation	Pesticide	Days After Application	ESTI	aHI	EDI	HQ	
						Korea	EU
Fruit powder	Sulfoxaflor	22 d	6.00 × 10^−6^	2.40 × 10^−5^	4.95 × 10^−6^	9.90 × 10^−5^	1.24 × 10^−4^
		15 d	7.50 × 10^−6^	3.00 × 10^−5^	6.00 × 10^−6^	1.20 × 10^−4^	1.50 × 10^−4^
		8 d	1.05 × 10^−5^	4.20 × 10^−5^	9.00 × 10^−6^	1.80 × 10^−4^	2.25 × 10^−4^
	Flupyradifurone	22 d	4.50 × 10^−5^	3.00 × 10^−5^	3.41 × 10^−5^	4.37 × 10^−4^	5.32 × 10^−4^
		15 d	5.70 × 10^−5^	3.80 × 10^−4^	4.40 × 10^−5^	5.63 × 10^−4^	6.87 × 10^−4^
		8 d	4.35 × 10^−5^	2.90 × 10^−4^	4.20 × 10^−5^	5.38 × 10^−4^	6.56 × 10^−4^
Fruit extract	Sulfoxaflor	22 d	2.00 × 10^−4^	8.00 × 10^−4^	1.65 × 10^−4^	3.30 × 10^−3^	4.13 × 10^−3^
		15 d	2.50 × 10^−4^	6.60 × 10^−5^	2.00 × 10^−4^	4.00 × 10^−3^	5.00 × 10^−3^
		8 d	3.50 × 10^−4^	1.26 × 10^−4^	3.00 × 10^−4^	6.00 × 10^−3^	7.50 × 10^−3^
	Flupyradifurone	22 d	1.50 × 10^−3^	1.86 × 10^−4^	1.14 × 10^−3^	1.46 × 10^−2^	1.77 × 10^−3^
		15 d	1.90 × 10^−3^	2.70 × 10^−3^	1.47 × 10^−3^	1.88 × 10^−2^	2.29 × 10^−2^
		8 d	1.45 × 10^−3^	3.02 × 10^−3^	1.40 × 10^−3^	1.79 × 10^−2^	2.19 × 10^−2^
Leaf powder	Sulfoxaflor	22 d	1.65 × 10^−5^	4.16 × 10^−3^	4.95 × 10^−5^	9.90 × 10^−5^	1.24 × 10^−4^
		15 d	3.15 × 10^−5^	6.60 × 10^−5^	6.00 × 10^−5^	1.20 × 10^−4^	1.50 × 10^−4^
		8 d	4.65 × 10^−5^	1.26 × 10^−4^	9.00 × 10^−6^	1.80 × 10^−4^	2.25 × 10^−4^
	Flupyradifurone	22 d	4.05 × 10^−4^	1.86 × 10^−4^	3.41 × 10^−5^	4.37 × 10^−4^	5.32 × 10^−4^
		15 d	4.53 × 10^−4^	2.70 × 10^−3^	4.40 × 10^−5^	5.63 × 10^−4^	6.87 × 10^−4^
		8 d	6.24 × 10^−4^	3.02 × 10^−3^	4.20 × 10^−5^	5.38 × 10^−4^	6.56 × 10^−3^

ESTI: estimated short-term intake; aHI: acute hazard index; EDI: estimated daily intake; HQ: Hazard Quotient. EU: European Union

**Table 6:** Pesticidal efficacy against two hemipteran insect pests at 7 days post-application.

Insect Pest	Pesticide	Survival Rate (Mean ± SD, %)	Control Efficacy (%)
*Aphis citricidus*	Sulfoxaflor WG	0	100
	Flupyradifurone SL	5.77 ± 1.57	96.00 ± 1.09
	Control	143.9 ± 7.41	-
Spotted Lanternfly	Sulfoxaflor WG	0	100
	Flupyradifurone SL	8.07 ± 3.07	91.9 ± 3.07
	Control	100	-

**Table 7:** Pre-harvest interval (PHI) and dissipation half-life of sulfoxaflor and flupyradifurone on crops.

Pesticide	Crop	PHI (Days)	Dissipation Half-Life (*t*_1_/_2_, Days)	Residue Level at PHI (mg/kg)	Reference
Sulfoxaflor	*C. tricuspidata* (fruit)	7	10.78	0.078 (predicted)	This study
Sulfoxaflor	*C. tricuspidata* (leaf)	7	3.84	0.546 (predicted)	This study
Sulfoxaflor	Cucumber	-	1.6–2.9	-	[20]
Sulfoxaflor	Spinach	21	3.83	0.25	[21]
Sulfoxaflor	Korean cabbage	14	3.54	0.31	[21]
Sulfoxaflor	Olive (fruit)	7	14.7	0.74	[18]
Flupyradifurone	*C. tricuspidata* (fruit)	7	14.02	0.412 (predicted)	This study
Flupyradifurone	*C. tricuspidata* (leaf)	7	12.96	5.213 (predicted)	This study
Flupyradifurone	Tea leaves	7	1.6	2.0	[16]
Flupyradifurone	Ginseng plant	21	4.5–7.9	0.272–1.835	[25]

PHI: Pre-harvest Interval.

## Data Availability

The original contributions presented in this study are included in the article/Supplementary Materials. Further inquiries can be directed to the corresponding author.

## Supplementary Materials

The following supporting information can be downloaded at https://www.mdpi.com/article/10.3390/toxics14020117/s1. Figure S1: Chromatograms depicting (A) sulfoxaflor in *Cudrania tricuspidata* leaves (a: sulfoxaflor (stereoisomer), b: sulfoxaflor (stereoisomer), c: X11719474, d: X1172061), (B) Flupyradifurone in *C. tricuspidata* leaves (a: flupyradifurone, b: 6-chloronicotinic acid, c: difluoroethyl-amino-furanone), and (C) Difluoroacetic acid, flupyradifurone metabolite, in *C. tricuspidata* leaves (A–C). The concentration of respective matrix-matched standards was 1.0 mg/kg; Figure S2. Dissipation kinetics of sulfoxaflor in (A) fruits and (B) leaves, and of flupyradifurone in (C) fruits and (D) and leaves; Table S1: Separation conditions for Sulfoxaflor and its metabolites; Table S2: Separation conditions for Flupyradifurone, 6-chloronicotinic acid, and difluororoethyl-amino-furanone; Table S3: Separation conditions for difluoroacetic acid; Table S4. Calibration equation for sulfoxaflor and flupyradifurone in fruits and leaves.
