# Supplementary material for: Sulfoxaflor and Flupyradifurone: Efficacy, Residue Dynamics, and Dietary Risk Assessment in Cudrania tricuspidata
Source: Toxics. 2026 Jan 26;14(2):117. doi: 10.3390/toxics14020117 (PMC12944189; doi:10.3390/toxics14020117)
Supplement: Supplementary file 1 [file toxics-14-00117-s001.zip › Supplementary Materials.pdf]

Supplementary Materials

Sulfoxaflor and Flupyradifurone: Efficacy, Residue Dynamics, and Dietary Risk Assessment of in *Cudrania tricuspidata*

Junheon Kim, Eunji Yu

Table S1. Separation conditions for Sulfoxaflor and its metabolites

Mobile phase				
	Time	Acetonitrile (%)	Water (%)	Formic acid (%)
	0.01	5	95	0.1
	1.00	5	95	0.1
	2.00	30	70	0.1
	8.00	30	70	0.1
	9.00	5	95	0.1
	12.00	5	95	0.1
Flow rate	0.3 mL/min			
Polarity	Positive			

Table S2. Separation conditions for Flupyradifurone, 6-chloronicotinic acid, and difluororoethyl-amino-furanone

Mobile phase				
	Time	Acetonitrile (%)	Water (%)	Formic acid (%)
	0.01	0	100	0.1
	1.00	0	100	0.1
	4.0	70	30	0.1
	6.0	70	30	0.1
	7.0	0	100	0.1
	10.0	0	100	0.1
Flow rate	0.3 mL/min			
Polarity	Positive			

Table S3. Separation conditions for difluoroacetic acid

Mobile phase				
	Time	Acetonitrile (%)	Water (%)	Formic acid (%)
	0.01	100	0	0.1
	1.50	100	0	0.1
	2.50	40	60	0.1
	4.00	40	60	0.1
	5.00	100	0	0.1
	8.00	100	0	0.1
Flow rate	0.4 mL/min			
Polarity	Negative			

Table S4. Calibration equation for sulfoxaflor and flupyradifurone in fruits and leaves

Pesticide	Fruits		Leaves	
	Linearity	R^2	Linearity	R^2
Sulfoxaflor				
Sulfoxaflor	Y = 2,383,399.1783x + 11,205.7506	0.9998	Y = 2,422,419.0240x + 3,795.2810	0.9990
X11719474	Y = 1,132,638.5162x + 7.855.3359	0.9992	Y = 1,160,930.3125x + 4,963.5047	0.9999
X11721061	Y = 986,666.1009x + 2,990.5059	0.9993	Y = 1,076,819.6807x + 1,209.6640	0.9998
Flupyradifurone				
Flupyradifurone	Y= 385,194.8968x - 1,629.0820	0.9995	Y = 326,50.9328x - 834.8018	0.9997
6-Chloronicotinic acid	Y= 230,100.9966x - 1,189.8643	0.9998	Y = 338,639.8801x - 173.3285	0.9996
Difluoroethyl-amino-furanone	Y= 180,502.2182x - 763.1815	0.9997	Y = 281,141.9568x - 750.5286	0.9999
Difluoroacetic acid	Y= 62,130.1408x - 104.8727	0.9990	Y = 66,617.5748x + 368.7929	0.9983

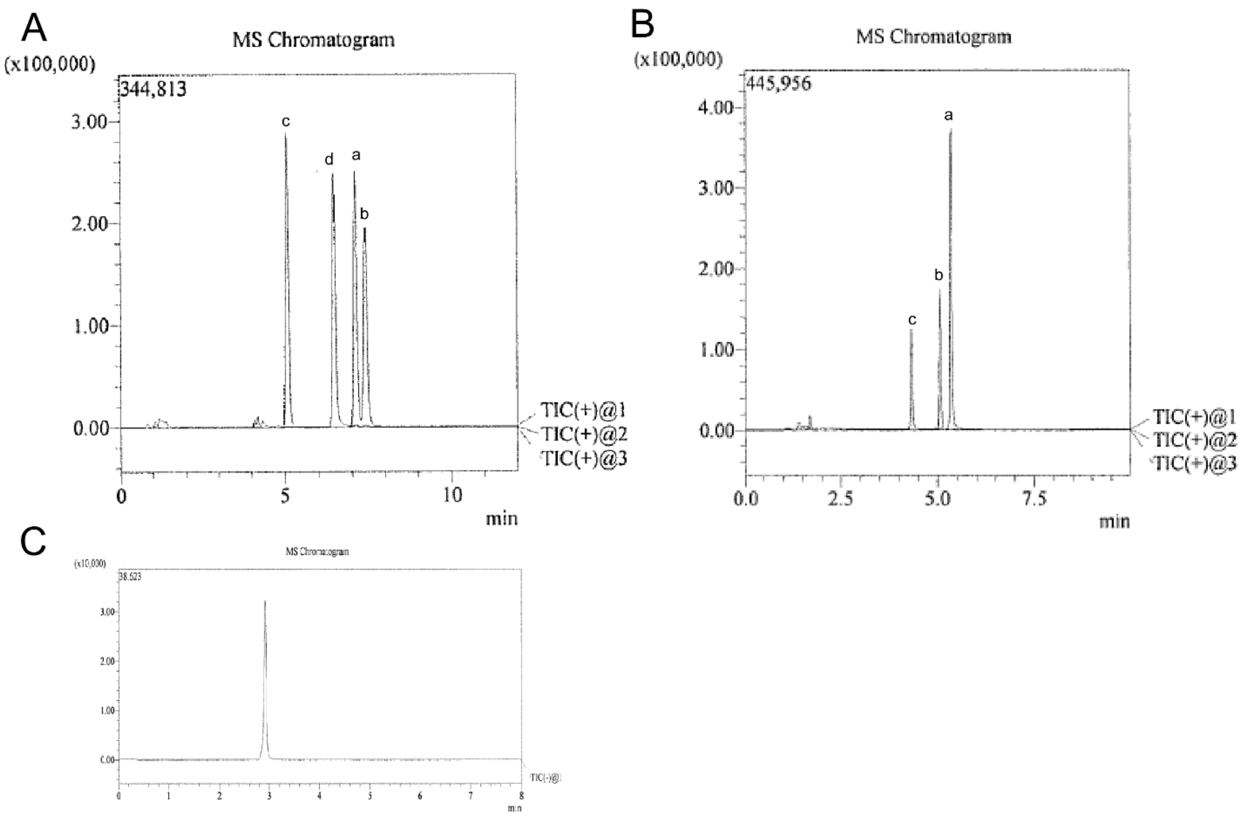

Figure S1. (A–C) Chromatograms depicting (A) sulfoxaflor in *Cudrania tricuspidata* leaves (a: sulfoxaflor (stereoisomer), b: sulfoxaflor (stereoisomer), c: X11719474, d: X1172061), (B) Flupyradifurone in *C. tricuspidata* leaves (a: flupyradifurone, b: 6-chloronicotinic acid, c: difluoroethyl-amino-furanone), and (C) Difluoroacetic acid, flupyradifurone metabolite, in *C. tricuspidata* leaves. (A–C) The concentration of respective matrix-matched standards was 1.0 mg/kg.

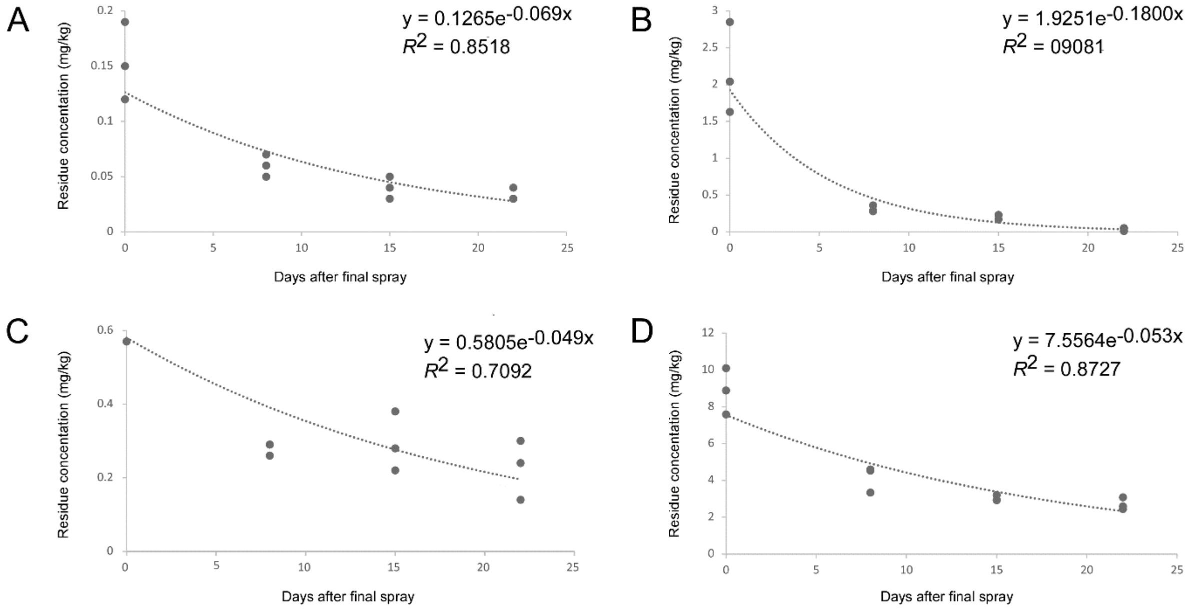

Figure S2. Dissipation kinetics of sulfoxaflor in (A) fruits and (B) leaves, and of flupyradifurone in (C) fruits and (D) and leaves
